# Anatomical Lens Position Predictability for a Capsulotomy-Fixated Intraocular Lens in Femtosecond Laser-Assisted Cataract Surgery

**DOI:** 10.3390/jcm14176151

**Published:** 2025-08-30

**Authors:** Colya N. Englisch, Philip Wakili, André Messias, Peter Szurman, Annekatrin Rickmann, Clemens N. Rudolph, Anna Theresa Fröhlich, Berthold Seitz, Achim Langenbucher, Karl T. Boden

**Affiliations:** 1 Eye Clinic Sulzbach, Knappschaft Hospitals Saar, 66280 Sulzbach/Saar, Germany; 2 Department of Experimental Ophthalmology, Saarland University, 66421 Homburg/Saar, Germany; 3 Department of Ophthalmology, Otorhinolaryngology and Head and Neck Surgery, School of Medicine of Ribeirão Preto, University of São Paulo, São Paulo 05508-220, Brazil; 4 Klaus Heimann Eye Research Institute (KHERI), 66280 Sulzbach/Saar, Germany; 5 Department of Ophthalmology, Saarland University Medical Center, 66421 Homburg/Saar, Germany

**Keywords:** cataract surgery, capsulotomy fixation, FEMTIS, TECNIS, ALP, ELP, prediction error, IOL power formulas

## Abstract

**Objectives**: To assess the anatomical lens position (ALP) predictability for a capsulotomy-fixated intraocular lens (IOL; FEMTIS FB-313, Teleon Surgical B.V) versus an intracapsular IOL (TECNIS 1-Piece, ZCB00V, Johnson & Johnson) implanted for age-related cataracts. **Methods**: Pre- and 3-month postoperative measurements from optical biometry and swept-source anterior segment OCT were analyzed. The lens position (*i.e.*, postoperatively, ALP) was defined as the distance between the corneal endothelium and the lens equator. Multivariate linear mixed-effects models assessed the influence of preoperative biometric parameters on ALP, prediction error (PE), and absolute PE (AbsPE). **Results**: A total of 45 FEMTIS eyes from 32 patients and 26 TECNIS eyes from 18 patients were included. Postoperatively, the anterior chamber depth (ACD) increased by 1.10 mm in FEMTIS eyes and 1.66 mm in TECNIS eyes, while the lens position decreased by 0.59 mm and 0.34 mm, respectively. ACD and lens thickness (LT) were the strongest ALP predictors (ACD: β = +0.70, *p* < 0.0001; LT: β = +0.37, *p* < 0.0001). Separate multivariate models demonstrated strong predictive performance, with the FEMTIS achieving R^2^ = 0.92 and the TECNIS IOL performing even better with R^2^ = 0.97. In the FEMTIS group, LT influenced the PE (*p* = 0.006) and ACD the AbsPE (*p* = 0.005). In the TECNIS group, ACD influenced the PE (*p* < 0.0001), while AbsPE was not significantly affected by biometric parameters or formulas. **Conclusions**: ALP can be reliably predicted from standard biometric data, although less accurately for the FEMTIS IOL. Its anterior capsulotomy fixation likely compromises refractive accuracy with formulas inherently designed and optimized for in-the-bag IOLs, emphasizing the need for fixation-specific formula adjustments and dedicated optimization in capsulotomy-fixated IOLs.

## 1. Introduction

Cataract surgery is among the most frequently performed ophthalmological procedures worldwide, with growing expectations for precise refractive outcomes. Despite advances in IOL design, measurement accuracy, and calculation methods, postoperative correction may still be necessary, often owing to inaccurate prediction of postoperative anatomical lens position (ALP) from preoperative data [1,2,3,4,5].

Target refraction calculations typically estimate a theoretical postoperative effective lens position (ELP) based on a thin lens model in untreated eyes [6,7,8], although ELP and ALP are not equivalent [9]. The ELP is a retrospective value derived from preoperative biometry, IOL power, and postoperative refraction in treated eyes, representing the correct position of a thin lens to achieve the necessary refraction [10,11]. By contrast, ALP can be measured postoperatively as the IOL’s equatorial plane position or estimated preoperatively using anterior chamber depth and central lens thickness [9,12]. However, the equatorial plane does not always match the principal plane, which requires knowledge of IOL design. This includes the power, shape factors, and material refractive index, which are often undisclosed by manufacturers [13]. In such cases, the principal plane can be approximated at half of the central IOL thickness.

IOL position is influenced by design, haptics, and placement technique. While most posterior chamber IOLs use in-the-bag fixation (*e.g.*, c-loop, plate haptic), some lenses utilize capsulotomy fixation, a distinct approach. The FEMTIS IOL (Teleon Surgical B.V., Spankeren, The Netherlands), designed for anterior capsulotomy fixation during femtosecond laser-assisted cataract surgery (FLACS), aims to enhance rotational stability and reduce tilt and decentration. Other lenses, such as the “bag-in-the-lens” IOL, Masket IOL, and 90F IOL, use similar approaches [14], but with limited availability or specific indications [15,16,17]. The FEMTIS IOL thus offers a unique platform for investigating ALP predictability in capsulotomy fixation. Its precise interaction with laser-created capsulotomies has shown good stability and visual outcomes [18]. Since most formulas assume a postoperative ALP within the capsular bag, capsulotomy fixation may alter this position, potentially increasing the prediction error (PE). While refractive outcomes have previously been assessed for the FEMTIS IOL [19], their reliance on subjective refraction limits accuracy and reproducibility [20,21], making ALP prediction a reliable alternative. Teshigawara *et al.* examined the FEMTIS IOL position within one month postoperatively [22], but since capsular contraction typically completes only after three months [23], later assessment is advised.

This study investigates whether preoperative biometric parameters can reliably predict the ALP of the FEMTIS IOL after FLACS, and compares this outcome with that of a standard one-piece IOL implanted during conventional cataract surgery. While previous work evaluated overall biometric predictability [19], this study specifically examines how individual preoperative factors relate to postoperative ALP and which of these factors contribute most to its estimation.

## 2. Materials and Methods

### 2.1. Study Patients

This retrospective study included adult patients who received either a FEMTIS or TECNIS IOL at Eye Clinic Sulzbach, Germany, between September 2020 and September 2024 with uneventful surgery and both pre- and post-operative anterior segment swept-source optical coherence tomography (OCT) taken at least three months after surgery. Exclusion criteria included lack of consent, age < 18, pregnancy, any anterior segment disease other than cataract, history of trauma, prior anterior segment surgery, corneal or lens-based refractive laser procedures, and preoperative corrected distance visual acuity (CDVA) > 0.2 logarithm of the minimal angle of resolution (LogMAR). All investigations were conducted in accordance with the tenets of the Declaration of Helsinki. All patients provided informed consent to participate in the study, and the local Institutional Review Board (Ethikkommission bei der Ärztekammer des Saarlandes) approved this study (175/14).

### 2.2. Preoperative and Postoperative Assessment

**Figure 1:**
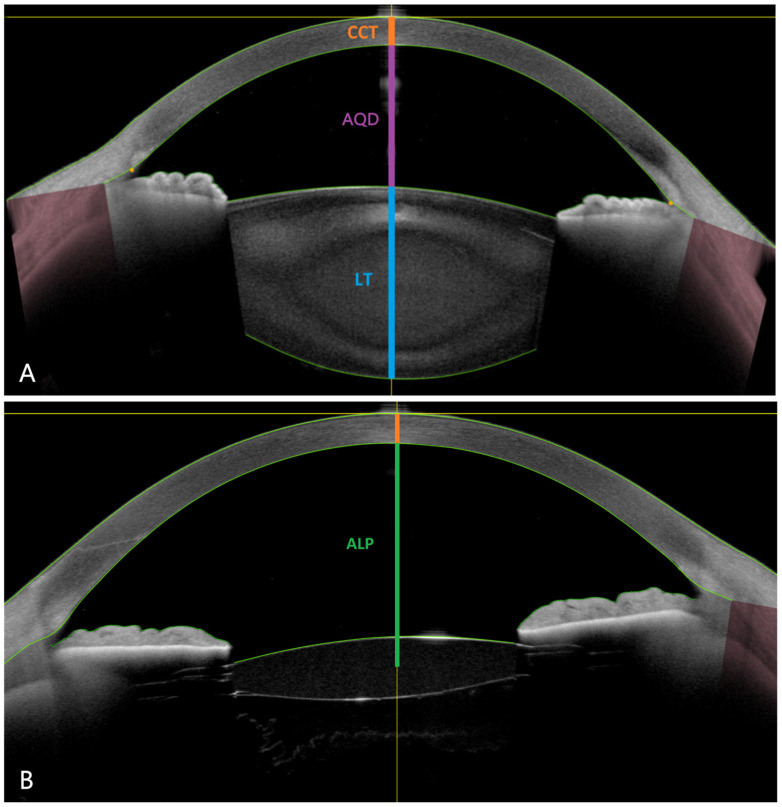
Anterior segment swept-source OCT scans (Anterion). (**A**) Preoperative central corneal thickness (CCT, orange), lens thickness (LT, blue), and aqueous depth (AQD, purple: the distance from the corneal endothelium to the anterior lens surface, shown for illustration purposes only; anterior chamber depth [ACD]~CCT + AQD). (**B**) Postoperative anatomical lens position (ALP, green) after FEMTIS IOL implantation.

Demographic data including age at surgery, sex, and right or left eye were recorded preoperatively. Approximately one week before surgery, patients received an entire ophthalmological examination. Optical biometry was performed preoperatively using an IOLMaster 700 (Carl Zeiss Meditec AG, Jena, Germany) and comprised AL: axial length (mm), LT: lens thickness (mm), ACD: anterior chamber depth (distance from the corneal epithelium to the anterior lens surface; mm), CCT: central corneal thickness (µm), WTW: white-to-white (*i.e.*, horizontal corneal diameter; mm), and K_m_: mean optical keratometry (D). Swept-source anterior-segment OCT (Anterion, Heidelberg Engineering GmbH, Heidelberg, Germany) was performed preoperatively and a minimum of three months postoperatively, a susceptible period for IOL position changes [23]. Preoperative measurements included AL, LT, ACD, CCT, and WTW (Figure 1A). Postoperative ALP was defined as the distance from the central corneal endothelium to half of the central IOL thickness (Figure 1B). CDVA and refraction were assessed subjectively pre- and a minimum of three months post-operatively.

### 2.3. FEMTIS FB-313 and TECNIS 1-Piece ZCB00V Intraocular Lenses

The FEMTIS IOL (Teleon Surgical B.V., Spankeren, The Netherlands) is a monofocal, one-piece posterior chamber lens made of hydrophilic acrylic, featuring an aspheric posterior surface and designed for fixation within a circular capsulotomy. Its optic measures 5.7 mm with an overall IOL diameter of 10.5 mm. Implantation involves positioning the larger leading haptic inside the capsular bag, while the four smaller trailing haptics are placed in front of the capsulotomy at 3, 6, 9, and 12 o’clock. The TECNIS 1-Piece IOL (ZCB00V, Johnson & Johnson, New Brunswick, NJ, USA) is also a monofocal, one-piece posterior chamber lens but is composed of hydrophobic acrylic, with a biconvex shape and an aspheric anterior surface. The lens has a 6.0 mm optic and a 13.0 mm overall diameter, with a C-loop haptics design.

### 2.4. Surgical Technique

While the TECNIS IOL was implanted during conventional cataract surgery, patients supplied with the FEMTIS IOL were treated with FLACS. In detail, the Ziemer LDV Z8 femtosecond laser (Ziemer Ophthalmic Systems, Port, Switzerland) was employed to create corneal incisions, perform the capsulotomy, and fragment the nucleus. Following laser treatment, the corneal incisions were gently opened, and the anterior chamber was filled with viscoelastic material. The free-floating capsulotomy was then extracted with forceps. Subsequent steps included hydrodissection and removal of both the nucleus and cortical material. The anterior chamber and capsular bag were then injected with cohesive viscoelastic (MINIVISC PLUS, Bohus BioTech AB, Strömstad, Sweden), after which the FEMTIS IOL was introduced into the capsular bag using the ACCUJECT injector system (Medicel AG, Altenrhein, Switzerland). Before sealing the paracenteses, the viscoelastic was carefully aspirated, and intracameral injections of acetylcholine, dexamethasone, and cefuroxime were administered.

### 2.5. Intraocular Lens Power Calculation Formulas

IOL power was estimated using nine calculation formulas. Whenever available, the manufacturer’s recommended lens constants were applied [19]; otherwise, constants were retrieved from the publicly accessible European Society of Cataract and Refractive Surgeons (ESCRS) IOL Calculator (https://iolcalculator.escrs.org/), based on data provided by IOLCon (www.IOLCon.org). For the FEMTIS IOL, calculations with the Haigis (a0 = 0.515; a1 = 0.400; a2 = 0.100) [24], SRK/T (a = 117.8) [8], Holladay 1 (SF = 0.97) [7], and Barrett Universal II (lens factor = 1.26) formulas were performed on the IOLMaster 700. Predictions from the Hoffer QST (pACD = 5.152) [25], EVO (a = 118.282), Pearl-DGS (a = 118.282), Cooke K6 (a = 118.282), and Kane (a = 118.282) formulas were obtained via the ESCRS IOL Calculator. Accordingly, for the TECNIS IOL, power calculations were carried out using the IOLMaster 700 for the Haigis (a0 = −1.356; a1 = 0.259; a2 = 0.249) [24], SRK/T (a = 119.4) [8], Holladay 1 (SF = 2.06) [7], and Barrett Universal II (lens factor = 2.04) formulas, and with the ESCRS IOL Calculator for the Hoffer QST (pACD = 5.8) [25], Cooke K6 (a = 119.3), EVO (a = 119.3), Pearl-DGS (a = 119.35), and Kane (a = 119.36) formulas. Target refraction was set between 0.0 and ±0.5 D for emmetropia and at −2.5 D to preserve myopia postoperatively. The PE was calculated as the difference between achieved and predicted spherical equivalent (SE) refraction. Fellow eyes were investigated independently without averaging their values.

### 2.6. Statistical Analysis

Normality was assessed using the Shapiro–Wilk test. Paired *t*-tests compared measurements from both biometers. Preoperative biometric and visual parameters were analyzed between both IOL groups using linear mixed-effects models, with eye as the unit and patient as a random intercept. Surgically-induced astigmatism (SIA) was evaluated using the Alpins vector K1–K3 method. Linear mixed-effects models included biometric variables (ACD, LT, AL, K_m_, and WTW) and formulas as fixed effects, with patient as a random effect, thus accounting for intra-individual correlation between fellow eyes. Interaction terms assessed whether biometric effects differed between both IOL types; separate models were used when indicated, and differences in variance were assessed using Levene’s test. Outcomes were the ALP, the PE, and the absolute PE (AbsPE). Model assumptions were verified using residual distributions. A two-tailed *p*-value of <0.05 was considered statistically significant. The term “significant” refers exclusively to statistical significance. Analyses were performed using JMP Pro (version 17.2, SAS Institute Inc., Cary, NC, USA).

## 3. Results

### 3.1. Demographics

A total of 45 eyes from 32 patients (19 male and 13 female) were included in the FEMTIS cohort, and 26 eyes from 18 patients (9 male and 9 female) in the TECNIS cohort. Mean age ± standard deviation (SD) was 71.1 ± 8.6 and 71.4 ± 8.6 years, respectively (*p* = 0.9). A total of 23 eyes were right, and 22 left in the FEMTIS cohort, and 12 eyes were right, and 14 left in the TECNIS cohort.

### 3.2. Preoperative FEMTIS vs. TECNIS Cohorts Biometry, Refraction, and Visual Acuity

Preoperative biometric parameters (AL, ACD, LT, K_m_, and WTW), as well as SE, cylinder, and IOL power, were comparable between eyes implanted with FEMTIS and TECNIS IOLs (Table 1). CDVA was slightly better in the FEMTIS group, though not clinically relevant, especially given the similar LT, indicating comparable cataract severity.

### 3.3. Preoperative Anterion vs. IOLMaster 700 Biometry

Preoperative AL measurements were virtually identical between both devices, with a mean difference (MD) of −0.0005 mm (*p* = 0.97). By contrast, IOLMaster 700 yielded a higher ACD, with a MD of +0.43 mm (3.17 ± 0.02 mm vs. 2.74 ± 0.02 mm; *p* < 0.0001); lower LT, with a MD of −0.067 mm (4.55 ± 0.02 mm vs. 4.61 ± 0.02 mm; *p* < 0.0001); and greater WTW, with a MD of +0.11 mm (12.00 ± 0.02 mm vs. 11.89 ± 0.02 mm; *p* < 0.0001). Despite these MDs, measurements from both devices showed strong correlations across all parameters (*r* > 0.9), supporting their internal consistency. All ALP measurements were obtained using the Anterion swept-source OCT in both IOL groups, ensuring consistency and eliminating inter-device variability in these investigations.

### 3.4. Postoperative Changes in Anterion Biometry

On average, ACD increased by 1.10 mm with the FEMTIS and 1.66 mm with the TECNIS IOL after phacoemulsification (*p* < 0.0001, for both). The corneal endothelium–lens equator distance (*i.e.*, postoperatively, ALP) decreased by 0.59 mm and 0.34 mm, respectively (*p* < 0.0001, for both). Postoperative CCT showed minor, nonsignificant reductions of 3.33 µm (*p* = 0.2) and 3.96 µm (*p* = 0.1). In turn, LT decreased by 3.45 mm and 3.85 mm (*p* < 0.0001, for both), AL by 0.09 mm (*p* = 0.0004) and 0.20 mm (*p* < 0.0001), and WTW by 0.05 mm (*p* = 0.02) and 0.02 mm (*p* = 0.03), respectively. 

Notably, based on the SDs of each group (FEMTIS: 0.31 mm; TECNIS: 0.32 mm) and a pooled SD of 0.41 mm, a statistical power of 94% was achieved to detect a between-group difference of 0.35 mm in ACD change corresponding to Cohen’s d = 0.86.

### 3.5. Influence of ACD, LT, AL, K_m_, WTW, IOL Power, and Type on the Anatomical Lens Position

Overall, the model showed strong predictive performance for ALP (R^2^*_adjusted_* = 0.94; root-mean-squared error (RMSE) = 0.1). ACD and LT were the strongest predictors, with higher values linked to a more posterior IOL position (ACD: β = +0.70, *p* < 0.0001; LT: β = +0.37, *p* < 0.0001). WTW showed a trend toward significance (β = +0.13, *p* = 0.059), indicating a potential contribution to deeper lens positioning. AL (β = +0.08, *p* = 0.4), K_m_ (β = +0.05, *p* = 0.2), and IOL power (β = +0.02, *p* = 0.5) were not significantly associated with ALP. By contrast, IOL type had a significant effect, with FEMTIS IOLs positioned, on average, 0.16 mm more anterior than TECNIS, independent of ocular anatomy (β = −0.16, *p* = 0.0002).

### 3.6. Predicting Anatomical Lens Position Using ACD and LT for Two IOL Designs

The separate multivariate linear model for the FEMTIS IOL achieved an adjusted R^2^ of 0.92 and a RMSE of 0.11 mm. For the TECNIS IOL, model performance was even stronger, with an adjusted R^2^ of 0.97 and a RMSE of 0.07 mm. Larger preoperative ACD and LT values predicted a more posterior ALP for both IOLs:

*ALP_FEMTIS_* = −0.333 + 0.747 × *ACD* + 0.371 × *LT*

**Figure 2:**
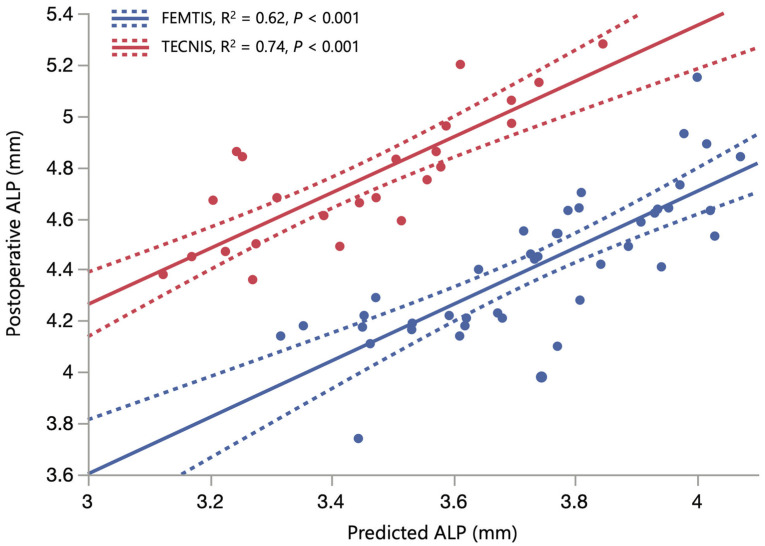
Scatter plots illustrating the relationship between measured anatomical lens position (ALP, y-axis, mm) and ALP predicted by the adjusted model (x axis, mm) for FEMTIS (blue) and TECNIS (red) intraocular lenses (IOLs). The symbols represent individual data points; the lines indicate linear fits by group (solid) and the 95% confidence intervals for the fits (dotted).

*ALP_TECNIS_* = −0.636 + 0.751 × *ACD* + 0.370 × *LT*

where ALP is the predicted anatomical lens position (mm), ACD the preoperative anterior chamber depth (mm), and LT the preoperative lens thickness (mm). Figure 2 illustrates the relationship between measured and predicted ALP, with the TECNIS model showing a higher adjusted R^2^ (0.74) than the FEMTIS model, with an R^2^ of 0.62 (Figure 2). Notably, the SD of the models’ residuals was higher in the FEMTIS group (0.174 mm) than in the TECNIS group (0.141 mm) (*p* = 0.4). Although nonsignificant, this might indicate greater variability in ALP when using an anterior capsulotomy-fixated IOL.

### 3.7. FEMTIS vs. TECNIS Postoperative Visual and Refractive Outcomes

Postoperative CDVA outcomes were excellent and comparable between cohorts (β = +0.023; *p* = 0.6). The mean postoperative CDVA was 0.13 ± 0.14 LogMAR for FEMTIS eyes (≈ 20/27 Snellen) and 0.10 ± 0.15 LogMAR for TECNIS eyes (≈ 20/26 Snellen), with most eyes achieving between 20/30 and 20/15. Mean SIA was nonsignificantly higher in the FEMTIS cohort (0.50 ± 0.57 D) compared with the TECNIS cohort (0.42 ± 0.35 D, *p* = 0.6). Given the small effect size and limited impact on visual acuity, these findings were not further explored in a power vector analysis. Notably, the FEMTIS cohort exhibited a slightly more hyperopic postoperative average SE (β = +0.21 D; *p* = 0.04).

### 3.8. FEMTIS vs. TECNIS Signed and Absolute Prediction Error

**Figure 3:**
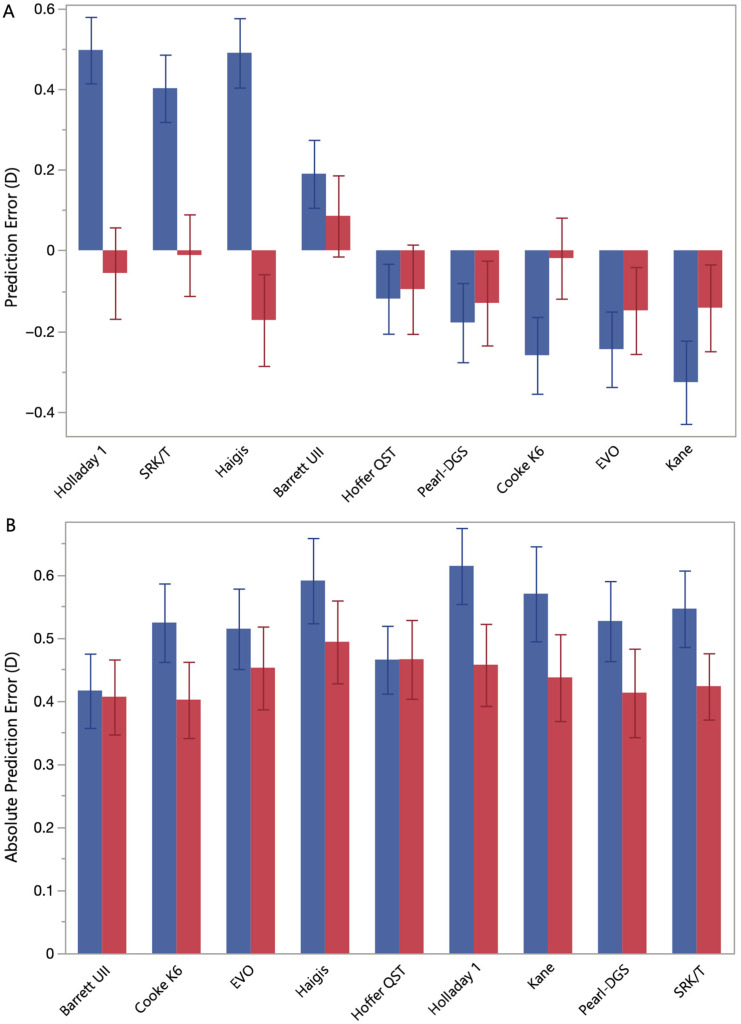
(**A**) Signed (PE) and (**B**) absolute (AbsPE) prediction error means (columns) with standard errors (error bars) for the FEMTIS (blue) and TECNIS (red) intraocular lenses (IOLs) for each formula.

Classical formulas such as Haigis, SRK/T, and Holladay 1 exhibited mean hyperopic shifts of +0.49 D, +0.40 D, and +0.50 D, respectively, in the FEMTIS group, compared with −0.17 D, −0.01 D, and −0.06 D in the TECNIS group (Figure 3A). Modern formulas such as Kane (−0.33 D vs. −0.14 D), EVO (−0.24 D vs. −0.15 D), and Pearl-DGS (−0.18 D vs. −0.13 D) yielded myopic shifts in both groups (Figure 3A). A significant interaction between IOL type and formula was observed (*p* < 0.0001): in the FEMTIS group, Haigis showed an additional +0.66 D in PE compared with TECNIS (*p* < 0.0001), Holladay 1 (+0.55 D, *p* < 0.0001), SRK/T (+0.41 D, *p* < 0.0001), and Barrett Universal II no meaningful difference (+0.10 D, *p* = 0.8), whereas Kane (−0.18 D, *p* < 0.0001), Cooke K6 (−0.24 D, *p* < 0.0001), EVO (−0.10 D, *p* < 0.0001), and Pearl-DGS (−0.05 D, *p* = 0.0005) showed larger myopic shifts.

For AbsPE, the interaction between IOL type and formula was not significant (*p* = 0.7), but mean values were higher for the FEMTIS cohort (0.54 ± 0.05 D) than for the TECNIS cohort (0.44 ± 0.06 D, *p* = 0.2) (Figure 3B). Among the formulas, Barrett Universal II, in contrast, achieved the lowest AbsPE of 0.41 ± 0.05 D, significantly outperforming Haigis (*p* = 0.01), with no other pairwise comparisons reaching significance (Figure 3B).

### 3.9. Influence of LT and ACD on Signed Prediction Error

Overall, LT significantly influenced the PE (*p* = 0.01), indicating that thicker lenses tended to yield more hyperopic outcomes, whereas ACD, AL, and WTW showed no significant associations. In the FEMTIS group, LT remained a significant predictor (*p* = 0.006), whereas ACD only showed a borderline effect (*p* = 0.09). In contrast, ACD was the strongest predictor in the TECNIS group (*p* < 0.0001), with deeper anterior chambers associated with a myopic shift, while LT showed only a marginal influence (*p* = 0.08). 

### 3.10. Influence of LT and ACD on Absolute Prediction Error

Overall, shallower anterior chambers were significantly associated with higher AbsPE (*p* = 0.003), whereas LT showed only a nonsignificant trend (*p* = 0.2). In the mixed-effects model across all formulas and IOL types, ACD also had a significant effect on AbsPE (*p* = 0.002) without significant formula interactions (*p* = 0.3). By contrast, LT effects were formula-specific; AbsPE increased in eyes with thicker lenses for Barrett Universal II (+0.25 D, *p* = 0.007), Holladay 1 (+0.23 D, *p* = 0.02), Haigis (+0.19 D, *p* = 0.047), EVO (+0.27 D, *p* = 0.003), and Kane (+0.26 D, *p* = 0.005) formulas. Within the FEMTIS group, ACD remained an independent predictor (*p* = 0.005), with greater preoperative depth improving prediction accuracy, whereas LT showed borderline significance (*p* = 0.07). In the TECNIS group, neither biometric parameter nor formula significantly influenced AbsPE, indicating a more uniform performance across formulas.

## 4. Discussion

Prediction of the postoperative IOL position remains one of the primary challenges in optimizing refractive outcomes after cataract surgery [10,13]. This issue becomes even more complex for novel lens designs such as the capsulotomy-fixated FEMTIS IOL, which deviate from conventional in-the-bag fixation assumptions embedded in most IOL power calculation formulas.

In this study, we found that preoperative ACD and LT were independent predictors of postoperative ALP for both IOL types, accounting for 92% and 97% of ALP variance in the FEMTIS and TECNIS groups, respectively, demonstrating predictive performance, even for nontraditional fixation techniques. Consistent with its capsulotomy-fixated design, which anchors the optic more anteriorly to traditional in-the-bag IOLs, the FEMTIS IOL was measured, on average, 0.16 mm more anterior than the TECNIS IOL. When examining refractive outcomes, clear differences emerged between the two lenses. The FEMTIS IOL demonstrated slightly greater variability and a trend toward higher AbsPE values, reflecting the mismatch between its fixation behavior and the assumptions inherent in existing formulas, as previously proposed [19]. Theoretical formulas such as the Haigis, SRK/T, and Holladay 1 showed hyperopic shifts in the FEMTIS cohort and slight myopic shifts in the TECNIS cohorts. Modern formulas including Kane, EVO, and Pearl-DGS produced mild myopic shifts in both cohorts. A closer look at biometric influences revealed that ACD played a consistent role in refractive accuracy overall. Shallower anterior chambers were associated with higher AbsPE across all formulas, indicating reduced precision in these eyes. This aligns with the general understanding that anterior segment anatomy can affect ELP predictability, also in modern formulas that incorporate ACD into the calculation. By contrast, LT had a formula-dependent effect. For the Barrett Universal II, Holladay 1, Haigis, EVO, and Kane formulas, thicker lenses were linked to increased AbsPE. Interestingly, in FEMTIS eyes, LT was significantly associated with the direction of refractive error, while ACD primarily affected the variability. In TECNIS eyes, however, ACD was the main predictor of the direction of refractive outcomes, while variability was less influenced by preoperative biometry. This might indicate an increased sensitivity of formula performance to anatomical variation in capsulotomy-fixated designs.

Overall, these results highlight that while ALP can be robustly predicted from standard biometric inputs, refractive predictability still depends on how well current formulas account for the unique fixation and behavior of each IOL design. For TECNIS, this alignment appears to be well-established; for FEMTIS, refinement is needed. This aligns with the conclusions of Shajari and colleagues, who, however, did not investigate the influence of preoperative biometry on ALP predictability and formula PEs [26]. In a previous study, we showed that even with accordingly optimized constants, refraction prediction is insufficient with regard to the high outcome expectations in FLACS [19]. 

Although our study provides important insights, especially given the limited data on ALP in both conventional and capsulotomy-fixated IOLs, it has limitations. The sample size was modest, though post hoc power analysis confirmed adequacy. Extreme anatomical variations (*e.g.*, very short or long AL) were under-represented. Only eyes with uneventful surgeries and good preoperative CDVA were included, limiting generalizability but aligning with typical use in patients with favorable ocular conditions and high refractive expectations. A high proportion of bilateral implantations introduced potential within-subject correlation, which was controlled using linear mixed-effects models. Cohort differences in ALP or refractive outcome may, though unlikely, partly reflect the laser capsulotomy performed only in the FEMTIS group, not solely IOL design. Overall, future work should investigate ALP predictability using established and optimized constants for anterior capsulotomy fixation. Beyond optimizing constants, purpose-specific powered prospective studies are needed to develop or refine IOL power formulas specifically for anterior capsulotomy-fixated designs, ideally based on datasets that include manufacturer-disclosed lens design parameters.

## 5. Conclusions

Taken together, ALP can be reliably predicted for both IOLs using standard biometric parameters. The more anterior ALP observed for the FEMTIS IOL relies on its anterior capsulotomy fixation. While this design may improve positional stability [18,22], it challenges the refractive accuracy of current IOL power formulas, which are primarily conceived and optimized for conventional in-the-bag placement. This underscores the importance of formula customization based on fixation type and highlights the need for dedicated optimization in capsulotomy-fixated IOLs.

## Figures and Tables

**Table 1:** Preoperative biometry using the IOLMaster 700, refraction, corrected distance visual acuity (CDVA), and IOL power for the FEMTIS and TECNIS intraocular lenses (IOLs). Data are reported as least-squares means ± standard error, adjusted for within-subject correlation using linear mixed-effects models. * indicates statistical significance.

Parameter	FEMTIS IOL	TECNIS IOL	*p*
**Axial Length (mm)**	23.52 ± 0.22	23.91 ± 0.29	0.3
**Anterior Chamber Depth (mm)**	3.13 ± 0.07	3.16 ± 0.10	0.8
**Lens Thickness (mm)**	4.57 ± 0.07	4.59 ± 0.10	0.8
**Mean Keratometry (D)**	42.91 ± 0.24	42.54 ± 0.32	0.4
**White to White (mm)**	12.05 ± 0.07	11.90 ± 0.10	0.2
**Spherical Equivalent (D)**	0.21 ± 0.81	−0.74 ± 1.17	0.5
**Cylinder (D)**	−1.30 ± 0.17	−1.63 ± 0.24	0.3
**CDVA (LogMAR)**	0.16 ± 0.01	0.19 ± 0.01	0.04 *
**IOL Power (D)**	20.23 ± 0.63	21.44 ± 0.84	0.3

## Data Availability

The original contributions presented in this study are included in the article. Further inquiries can be directed to the corresponding authors.

## References

[1] Langenbucher A., Hoffmann P., Cayless A., Bolz M., Wendelstein J., Szentmary N. (2024). Impact of uncertainties in biometric parameters on intraocular lens power formula predicted refraction using a Monte-Carlo simulation. Acta Ophthalmol..

[2] Norrby S. (2008). Sources of error in intraocular lens power calculation. J. Cataract. Refract. Surg..

[3] Olsen T. (2007). Calculation of intraocular lens power: A review. Acta Ophthalmol. Scand..

[4] Plat J., Hoa D., Mura F., Busetto T., Schneider C., Payerols A., Villain M., Daien V. (2017). Clinical and biometric determinants of actual lens position after cataract surgery. J. Cataract. Refract. Surg..

[5] Chang J., Wang L., Jiang C., Song Z., Lu P. (2024). Predicting the postoperative intraocular lens position based on IOL Master 700 biometry, compared with results from the anterior segment analysis system. Graefes Arch. Clin. Exp. Ophthalmol..

[6] Hoffer K.J. (1993). The Hoffer Q formula: A comparison of theoretic and regression formulas. J. Cataract. Refract. Surg..

[7] Holladay J.T., Prager T.C., Chandler T.Y., Musgrove K.H., Lewis J.W., Ruiz R.S. (1988). A three-part system for refining intraocular lens power calculations. J. Cataract. Refract. Surg..

[8] Retzlaff J.A., Sanders D.R., Kraff M.C. (1990). Development of the SRK/T intraocular lens implant power calculation formula. J. Cataract. Refract. Surg..

[9] Schroder S., Langenbucher A. (2018). Relationship between effective lens position and axial position of a thick intraocular lens. PLoS ONE.

[10] Langenbucher A., Szentmary N., Cayless A., Wendelstein J., Hoffmann P. (2022). Prediction of the axial lens position after cataract surgery using deep learning algorithms and multilinear regression. Acta Ophthalmol..

[11] Savini G., Taroni L., Hoffer K.J. (2020). Recent developments in intraocular lens power calculation methods-update 2020. Ann. Transl. Med..

[12] Olsen T. (2006). Prediction of the effective postoperative (intraocular lens) anterior chamber depth. J. Cataract. Refract. Surg..

[13] Olsen T., Cooke D.L., Findl O., Gatinel D., Koch D., Langenbucher A., Melles R.B., Yeo T.K. (2023). Surgeons need to know more about intraocular lens design for accurate power calculation. J. Cataract. Refract. Surg..

[14] Holland D., Rufer F. (2020). New intraocular lens designs for femtosecond laser-assisted cataract operations: Chances and benefits. Ophthalmologe.

[15] Tassignon M.J., De Groot V., Vrensen G.F. (2002). Bag-in-the-lens implantation of intraocular lenses. J. Cataract. Refract. Surg..

[16] Masket S., Fram N.R., Cho A., Park I., Pham D. (2018). Surgical management of negative dysphotopsia. J. Cataract. Refract. Surg..

[17] Dick H.B., Schultz T. (2014). Intraocular lens fixated in the anterior capsulotomy created in the line of sight by a femtosecond laser. J. Refract. Surg..

[18] Auffarth G.U., Friedmann E., Breyer D., Kaymak H., Holland D., Dick B., Petzold A., Shah S., Ladaria L.S., Garcia S.A. (2021). Stability and Visual Outcomes of the Capsulotomy-Fixated FEMTIS-IOL After Automated Femtosecond Laser-Assisted Anterior Capsulotomy. Am. J. Ophthalmol..

[19] Englisch C.N., Boden K.T., Messias A., Szurman P., Rickmann A., Muller L.J., Lorenz A.T., Seitz B., Langenbucher A., Wakili P. (2025). Refraction Predictability for a Capsulotomy-Fixated Intraocular Lens in Femtosecond Laser-Assisted Cataract Surgery. J. Refract. Surg..

[20] Hirnschall N., Farrokhi S., Amir-Asgari S., Hienert J., Findl O. (2018). Intraoperative optical coherence tomography measurements of aphakic eyes to predict postoperative position of 2 intraocular lens designs. J. Cataract. Refract. Surg..

[21] Shah R., Edgar D.F., Rabbetts R., Harle D.E., Evans B.J. (2009). Standardized patient methodology to assess refractive error reproducibility. Optom. Vis. Sci..

[22] Teshigawara T., Meguro A., Mizuki N. (2021). Relationship Between Postoperative Intraocular Lens Shift and Postoperative Refraction Change in Cataract Surgery Using Three Different Types of Intraocular Lenses. Ophthalmol. Ther..

[23] Wallace H.B., Misra S.L., Li S.S., McKelvie J. (2018). Predicting pseudophakic refractive error: Interplay of biometry prediction error, anterior chamber depth, and changes in corneal curvature. J. Cataract. Refract. Surg..

[24] Haigis W., Lege B., Miller N., Schneider B. (2000). Comparison of immersion ultrasound biometry and partial coherence interferometry for intraocular lens calculation according to Haigis. Graefes Arch. Clin. Exp. Ophthalmol..

[25] Taroni L., Hoffer K.J., Pellegrini M., Lupardi E., Savini G. (2023). Comparison of the new Hoffer QST with 4 modern accurate formulas. J. Cataract. Refract. Surg..

[26] Shajari M., Sonntag R., Niermann T., Holland D., Kohnen T., Priglinger S., Mayer W.J. (2020). Determining and Comparing the Effective Lens Position and Refractive Outcome of a Novel Rhexis-Fixated Lens to Established Lens Designs. Am. J. Ophthalmol..

